# Exosomes Secreted by Microglia During Virus Infection in the Central Nervous System Activate an Inflammatory Response in Bystander Cells

**DOI:** 10.3389/fcell.2021.661935

**Published:** 2021-08-13

**Authors:** Nhungoc Luong, Julie K. Olson

**Affiliations:** ^1^Department of Veterinary and Biomedical Sciences, University of Minnesota, Minneapolis, MN, United States; ^2^Department of Diagnostic and Biological Sciences, University of Minnesota, Minneapolis, MN, United States

**Keywords:** microglia, neuroinflammation, virus infection, demyelinating disease, exosome

## Abstract

Microglia become persistently infected during Theiler’s murine encephalomyelitis virus (TMEV) infection in the central nervous system (CNS) of susceptible mice. We have previously shown that microglia infected with TMEV become activated through the innate immune receptors to express type I interferons, cytokines, and chemokines. Persistent TMEV infection in the CNS promotes chronic neuroinflammation and development of demyelinating disease similar to multiple sclerosis. In the current studies, we wanted to determine whether TMEV-infected microglia secrete exosomes which contribute to neuroinflammation in the CNS thus promoting the development of demyelinating disease. Exosomes are vesicles containing RNA, DNA, and proteins that are released from one cell and taken up by another cell to facilitate communication between cells. These studies isolated exosomes secreted by microglia during TMEV infection *in vitro* as well as exosomes secreted by microglia during early TMEV infection in mice. These studies show that microglia secrete exosomes during TMEV infection which contain the viral RNA coding region. The exosomes secreted by microglia during TMEV infection can be taken up by uninfected bystander cells, including CNS resident microglia, astrocytes, and neurons. The viral RNA in the exosomes can be transferred to the bystander cells. In addition, the bystander cells that took up these exosomes were activated through the innate immune response to express type I interferons, IFNα and IFNβ, pro-inflammatory cytokines, IL-6, IL-12, and TNFα, and chemokines, CCL2. Most interestingly, exosomes secreted by microglia during early TMEV infection in mice activated an inflammatory response when transferred to the brains of naïve mice. These results show that exosomes secreted by microglia during early TMEV infection contain viral RNA and can activate uninfected bystander CNS cells to promote an inflammatory immune response. Thus, exosomes secreted by microglia during virus infection may promote viral persistence and neuroinflammation which contributes to the development of demyelinating disease.

## Introduction

Theiler’s murine encephalomyelitis virus (TMEV) is a natural mouse pathogen that can establish a persistent virus infection in the central nervous system (CNS). TMEV is a picornavirus which has a single positive-stranded RNA genome and has no envelope. TMEV infection of susceptible mice, such as SJL mice, establishes a persistent infection in the microglia/macrophage in the brain and spinal cord (Lipton et al., 1995; Olson et al., 2001). The persistent infection leads to the development of a chronic, progressive demyelinating disease beginning with clinical disease around 35–40 days post-infection (Lipton, 1975; Clatch et al., 1985). TMEV-induced demyelinating disease has been shown to be associated with an inflammatory immune response in the CNS and development of autoimmune CD4^+^ T cell response directed against myelin antigen, proteolipid protein, PLP_139__–__151_ (Canto et al., 2000; Neville et al., 2002). We have previously shown that the innate immune response to virus infection influences the development and progression of demyelinating disease (Bowen and Olson, 2009; Olson and Miller, 2009). TMEV-induced demyelinating disease has several immunological and pathological similarities to multiple sclerosis (MS) in humans (Dal Canto, 1975; Lipton et al., 1986). Microglia are the resident immune cells of the CNS that originate from the yolk sac during development. Microglia express innate immune receptors which enable them to rapidly respond to pathogens invading the CNS (Olson and Miller, 2004). Microglia become activated through the innate immune receptors to express cytokines, chemokines, and effector molecules. We have previously shown that TMEV infection of microglia leads to a rapid expression of type I interferons, IFNα and IFNβ (Olson et al., 2001). We have also shown that microglia infected with TMEV become activated to express pro-inflammatory cytokines, IL-1β, IL-6, IL-12, TNFα, chemokines, CCL2, CCL3, CCL5, and effector molecules, inducible nitric oxide (iNOS) (Dal Canto, 1975; Lipton, 1975; Clatch et al., 1985; Lipton et al., 1986; Canto et al., 2000; Olson et al., 2001; Neville et al., 2002; Olson and Miller, 2004, 2009; Bowen and Olson, 2009). TMEV-infected microglia also become activated antigen presenting cells that can present viral antigens and myelin antigens to CD4^+^ T cells (Olson et al., 2001). Exosomes are derived from microvesicular bodies (80–120 nm) within the cell and are then released from the cell. Exosomes secreted from one cell can fuse with target cells releasing the components of the exosome into the target cell, thus exosomes provide a means of communication between cells. Exosomes contain proteins, lipids, and nucleic acids, including mRNA, microRNA, and DNA (Janas et al., 2015). The exosome membranes resemble the lipid bilayer membrane containing exosome-specific markers such as tetraspanin (CD63, CD81) and syndecans (Simons and Raposo, 2009; Record et al., 2014). The exosomes structure allows them to securely transfer various materials across the blood-brain barrier and prevent the degradation from surrounding RNAse and proteases (Schneider and Simons, 2013). Recent studies have revealed the important role of exosomes in the pathogenesis of neurodegenerative diseases via the transfer of miRNAs, pathogenic, and misfolded proteins from rafts to recipient cells (Vella et al., 2008; Schneider and Simons, 2013). More recently, exosomes have been isolated from the sera of chronically infected hepatitis C virus (HCV) patients and shown to contain replication-competent viral RNA (Bukong et al., 2014). Further studies showed that exosome-packaged HCV could induce phenotype and cytokine profile switch in recipient macrophage (Banishree et al., 2017). Exosomes represent a novel pathway for communication between cells, thus exosomes secreted by microglia may play an important role in communication between cells in the CNS. Microglia have been shown to be infected during TMEV infection, thus we wanted to determine whether TMEV-infected microglia secrete exosomes which may contribute to persistent virus infection and inflammation in the CNS. Our studies showed that exosomes secreted from microglia during TMEV infection do not contain TMEV viral particles but do contain the viral RNA genome which can be transferred to uninfected CNS resident cells such as microglia, astrocytes, and neurons. More importantly, these exosomes activated bystander CNS cells to express type I interferons and pro-inflammatory cytokines and chemokines through innate immune receptor recognition of the viral RNA. Further, exosomes secreted by microglia in the brain during early TMEV infection contained viral RNA which could be transferred to naïve mice activating an inflammatory immune response in the recipient mice. The results from these studies suggest a new pathway via exosomes by which viral RNA can be transferred between cells during infection in the CNS independent of viral particles and by which neuroinflammation can be exacerbated during virus infection in the CNS.

## Materials and Methods

### Mice

Female SJL mice age 5–6 weeks were purchased from Envigo (Madison, WI, United States). Pregnant SJL/J mice (15–17 days) were purchased from Envigo. Neonatal SJL mice were used for primary cell isolation. The mice were housed at University of Minnesota Research Animal Resource Center accredited by the American Association for Accreditation of Laboratory Animal Care. The animals are handled according to University and Animal Care and Use Committee approved protocols.

### Isolation and Culture of CNS Cells

Isolation of primary glial cultures from neonatal mice was performed, as previously described (Olson et al., 2001). Briefly, brains were removed from 1 to 3 day old mice, and the meninges were removed. The left and right hemispheres of the brain were gently dissociated in a nylon mesh bag. The cells were resuspended in DMEM-F12 media (Lonza) supplemented with 10% FCS (Invitrogen Life Technologies) and 100 U/ml penicillin and 100 μg/ml streptomycin (Invitrogen Life Technologies). The cells were seeded in poly(D-lysine) (Sigma-Aldrich) coated tissue culture flasks and incubated at 37°C. After 10–14 days of incubation, microglia were removed from the astroglial layer by shaking the flasks on an orbital shaker for 24 h. The primary microglia were removed from the flask and placed in DMEM (Invitrogen Life Technologies) supplemented with 10% exosome free FCS and 3 ng/ml rGM-CSF (R&D Systems). The microglia were seeded in 24 well plates coated with poly(D-lysine). Astrocytes were cultured in supplemented DMEM-F12 media with exosome free FCS as described above. Microglia cultures were 98% pure as determined by CD11b staining. Astrocytes were greater than 95% pure based on expression of GFAP. For neurons, the brain tissue was dissociated with 0.25% trypsin for 15 min, and the cell suspension was placed in laminin coated flasks with B27 supplemented neurobasal media (Gibco, Invitrogen). Neuron cultures were 98% pure based on expression of NeuN. The microglia were transfected with siRNA specific for MyD88, Ticam1, or mitochondrial antiviral-signaling protein (MAVS), SMARTpool siRNA (5 μM), or siCONTROL (Dharmacon) using Dharmafect 4 following the protocol provided by Dharmacon.

### TMEV Infection

SJL female mice were intracranially injected with 2 × 10^6^ PFU of BeAn strain of TMEV. The BeAn strain 8386 of TMEV is propagated on baby hamster kidney cells (BHK-21) as previously described (Lipton, 1975; Clatch et al., 1985). Microglia were infected with the BeAn strain of TMEV at a multiplicity of infection of 5 in serum-limited DMEM for 24 h as previously described (Olson et al., 2001).

### Exosome Isolation and Analysis

Cell culture media was removed from the cells and centrifuged at 2,000 × *g* to remove cellular debris. Total exosome isolation reagent for cell culture (Invitrogen) was added to the supernatant (1:2 ratio) and incubated overnight at 4°C. The exosomes were pelleted the next day by centrifuging at 10,000 × *g* for 1 h per the protocol (Invitrogen). For isolating exosomes from brains, the brains were dissociated through a 70 μM filter into 3 ml Hank’s balanced salt solution. The homogenate was then centrifuged two times at 2,000 × *g* to remove cells and debris. The exosomes were isolated from the cleared homogenate with the isolation reagent as described above. The exosomes were then incubated with antibody for CD11b conjugated to magnetic beads and separated on a column per manufacturer protocol (Miltenyi). To determine size of the exosomes, exosomes were resuspended in PBS and analyzed on a Nanosight N300 (Malvern). To determine purity, exosomes were imaged on a transmission electron microscope (TEM). Briefly, the exosomes were fixed with 2% glutaraldehyde for 1 h and then absorbed onto a glow discharged carbon-Formvar coated 200-mesh copper grids for 5 min. Grids were washed twice with water and stained with 2% uranyl acetate twice for 30 s. TEM imaging was performed on a Tecnai G2 F30 instrument. Exosomes were analyzed by flow cytometry for surface protein expression. Briefly, exosomes were incubated with aldehyde/sulfate latex beads (Invitrogen) per the manufacturer protocol. The exosomes were then incubated with fluorescently labeled antibodies for CD45, CD11b, CD63, I-As, CD80, and CD86. The exosomes were washed and analyzed on LSRII (BD). Florescent imaging of exosomes was conducted by labeling exosomes with carboxyfluorescein succinimidyl ester (2 μM) or SYTO RNA select (Thermo Fisher Scientific). Microglia were labeled with fluorescently labeled antibody for CD11b and imaged on Olympus confocal microscope. Isolated exosomes were also analyzed for CD63 by western blot (Bio-Rad) following lysis using Total Exosome RNA and protein isolation kit (Life Sciences). Additional protein analysis was conducted using mass spectrometry. Proteins were separated on 4–12% Bis-Tris gels (Invitrogen). Cysteine bonds were reduced and alkylated with 10 nM DTT in 50 mM ammonium bicarbonate and 55 mM iodoacetamide:50 mM NH_4_HCO_3_. Proteins were digested in 50 mM NH_4_HCO_3_, 5 mM CaCl_2_, 5 ng/μl typsin. The samples were eluted with 60:40 acetonitrile:H_2_O, 0.1% trifluoroacetic acid before analysis on Thermo Orbitrap Elite mass spectrometer at the University of Minnesota Center for Mass Spectrometry. To determine concentration of exosomes, Bradford assay was performed. Isolated exosomes were incubated with RNAse (100 ng/ml) (Thermo Fisher Scientific), or trypsin (1 mg/ml) (Mediatech) and pepsin (10 μg/ml) (Sigma) for 10 min at 37°C and then washed with PBS before analysis or adding to the cells. Isolated exosomes (100 μg) were added to cultured cells (1 × 10^6^) or injected intracranially (500 μg) into a mouse.

### Viral Plaque Assay

The exosomes were isolated and resuspended in serum-free DMEM. The supernatant from microglia cultures incubated with exosomes or infected with TMEV were collected and concentrated 100 fold. The brain was removed and homogenized in serum-free DMEM. BHK-21 cells were grown in a confluent monolayer prior to addition of the exosomes, microglia supernatants or homogenized tissue dilutions. The cells were incubated at room temperature for 1 h. The cells were overlaid with a 2% agar/DMEM solution and incubated at 34°C for 5 days. The cells were fixed with methanol and stained with crystal violet solution (0.12% crystal violet). The plaques were counted on each plate and multiplied by the dilution or the amount of homogenate added to the plate to determine the PFU/ml. The weight of the tissue (mg) and homogenate volume was then used to calculate PFU/mg.

### RNA Isolation and PCR Analysis

RNA was isolated from exosomes using the Total Exosome RNA and protein isolation kit (Life Sciences). RNA was isolated from microglia, astrocytes, and neurons using SV Total RNA Isolation kit which contains a DNAse reaction (Promega). RNA was isolated from brain tissue using TRIzol protocol followed by DNAse digestion (Thermo Fisher Scientific). First strand cDNA was generated from 1 μg of total RNA using oligo(dT)_12__–__18_ primers and Advantage for RT-PCR kit in a final volume of 100 μl (Clontech). Real-time PCR was conducted in triplicate with Rotor-Gene SYBR green RT-PCR kit (Qiagen). Briefly, 0.5 μM primers, 1× SYBR Green reagent, and 2 μl of cDNA were combined in 10 μl reactions. The primers for TMEV, cytokines, chemokines, and effector molecules were previously described (Dal Canto, 1975; Lipton, 1975; Clatch et al., 1985; Lipton et al., 1986; Canto et al., 2000; Olson et al., 2001; Neville et al., 2002; Olson and Miller, 2004, 2009; Bowen and Olson, 2009). Real time PCR was conducted on a Rotor-Gene Qiagen Q instrument using hot start with cycle combinations, 40 cycles: 95°C for 15 s; 60°C for 20 s; 72°C for 15 s, followed by a melt from 75 to 95°C. Quantitation of the mRNA was based on standard curves derived from cDNA standards for each primer pair. Positive and negative cDNA controls were used for each primer pair using cells known to express or not express the specific mRNA. Samples from different groups were normalized based on expression of β-actin. All samples were run in triplicate for each primer pair. Statistical analysis comparison between groups was determined by one way ANOVA and Bonferroni’s multiple comparison test (*p* < 0.001). PCR for viral genomes was conducted using cDNA in 25 μl reactions with TMEV primers (0.5 μM), dNTPs (200 μM), 1× reaction buffer, and Q5 high fidelity DNA polymerase (0.02 U/μl) (New England BioLabs) on Eppendorf Mastercycler with a hot start and 40 cycles: 95°C for 30 s; 60°C for 30 s; 72°C for 8 m, followed by a 20 m extension. PCR products were separated on 0.8% agarose gel with SYBR Safe (Thermo Fisher Scientific) and imaged.

### Statistics

Experiments were conducted in triplicate for each sample (real time PCR). All the experiments were independently repeated at least three or more times. The significant difference between control samples and experimental samples was determined using one way ANOVA and Bonferroni’s multiple comparison test (*p* < 0.001). The sample size of mice per group were calculated to provide 80% power at *p* < 0.05.

## Results

### Microglia Infected With TMEV Secrete Exosomes That Contain Viral RNA

**FIGURE 1:**
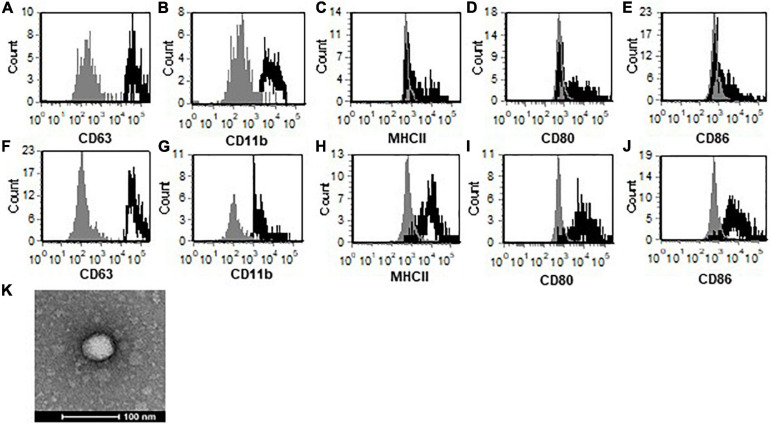
TMEV-infected microglia secrete exosomes that contain activation markers. Exosomes were isolated from uninfected **(A–E)** or TMEV infected microglia **(F–J)**. The exosomes were labeled with fluorescently labeled antibodies for CD63 **(A,F)**, CD11b **(B,G)**, MHC class II **(C,H)**, CD80 **(D,I)**, and CD86 **(E,J)**. The exosomes were analyzed by flow cytometry for expression of specific markers as shown in the black line compared to isotype control antibodies in the gray line. **(K)** Isolated exosomes were analyzed by transmission electron microscopy and determined to be 40–80 nm. One representative image is shown. These are representative graphs and images from one experiment of four independent repeated experiments.

Microglia secrete exosomes under normal conditions, therefore, we wanted to determine whether exosomes secreted by microglia during TMEV infection have altered contents. Primary microglia were infected with TMEV *in vitro*, and exosomes were isolated after 24 h. The isolation of exosomes was determined based on expression of exosome specific proteins such as CD63, shown by flow cytometry (Figure 1), and CD9, CD81, TSG101, Rab proteins, as determined by mass spectrometry (Supplementary Table 1). The exosomes also lacked the expression of Grp94, calnexin, cytochrome C, histones, and argonaute/RISC complex which are associated with other types of extracellular vesicles. The exosomes were further examined by TEM and Nanosight to determine purity and size with average size about 120 nm (Figure 1 and Supplementary Figure 1). These analyses confirm isolation and identification of exosomes for publication as determined by the International Society for Extracellular Vesicles Lötvall et al., 2014. Furthermore, the exosomes isolated from TMEV-infected microglia do not contain viral particles based on TEM analysis which showed no viral particles inside the exosomes or associated with exosomes, viral plaque assays with isolated exosomes which resulted in no plaques on BHK cells, mass spectrometric analysis of isolated exosomes which detected no viral proteins present in exosomes, and Nanosight particle size of exosomes which showed no particles smaller than 60 nm (TMEV is 30–40 nm) (Figure 1, Supplementary Figure 1, and Supplementary Table 1). Next, we wanted to determine whether exosomes secreted from microglia contain surface proteins similar to microglia. Exosomes isolated from both uninfected and infected microglia have CD11b on the surface (Figure 1). Exosomes from uninfected microglia have low levels of co-stimulatory molecules, CD80 and CD86, and do not have MHC class II. Interestingly, exosomes from TMEV-infected microglia have higher levels of co-stimulatory molecules and have MHC class II compared to exosomes from uninfected microglia.

**FIGURE 2:**
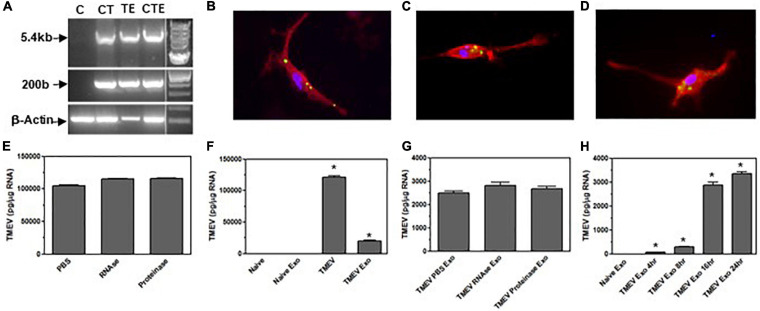
Exosomes from TMEV-infected microglia contain viral RNA that is transferred to bystander microglia. **(A)** Microglia were infected with TMEV and exosomes were isolated (lane TE). Microglia (1 × 10^6^) were uninfected (lane C), infected with TMEV (lane CT), or incubated with exosomes (100 μg) from TMEV-infected microglia (lane CTE). The cells were lysed 24 h later, RNA isolated, converted to cDNA, and used in PCR analysis with primers for TMEV long (5.4 kbp) or short (200 bp) products or with primers for β-actin. **(B)** Exosomes isolated from TMEV-infected microglia were fluorescently labeled with 2 μM CFSE (green). The exosomes were placed in culture with naive microglia for 2 h, and the microglia were fixed and incubated with fluorescently labeled antibody for CD11b (red). Exosomes isolated from TMEV-infected microglia were incubated with RNA stain (green) and placed on microglia for 2 h **(C)** or 4 h **(D)**. Microglia were fixed and incubated with fluorescently labeled antibody for CD11b (red). Cells were analyzed by confocal microscopy. **(E)** Exosomes from TMEV-infected microglia were isolated and control treated (PBS), RNAse treated, or proteinase treated. The RNA was isolated from the exosomes, converted to cDNA, and used in real time PCR with primers for TMEV. **(F)** Microglia were uninfected (naïve) or infected with TMEV. Microglia were incubated with exosomes isolated from TMEV-infected microglia or uninfected microglia. The microglia were lysed 24 h later, RNA isolated, converted to cDNA, and real time PCR conducted with primers for TMEV. **(G)** Exosomes were isolated from TMEV-infected microglia and control treated (PBS), RNAse treated or proteinase treated prior to putting the exosomes into culture with naïve microglia. The microglia were lysed 24 h later and analyzed by real time PCR for TMEV. **(H)** Exosomes were isolated from TMEV-infected microglia and placed on naïve microglia. After 4 h, the exosomes were removed and the cells were washed and incubated for an additional 0, 4, 12, or 20 h before being lysed and analyzed by real time PCR for TMEV. Significant difference was determined by one way ANOVA and Bonferroni’s multiple comparison test (*p < 0.001) based on expression by naïve microglia. These are representative graphs from one experiment of six independent repeated experiments.

Exosomes have been shown to contain RNA, therefore, we wanted to determine whether exosomes from TMEV-infected microglia contain viral RNA. The exosomes were isolated from TMEV-infected microglia and examined for viral RNA. First, primers for a short piece at the beginning of the viral genome were used to generate a 200 bp product which showed that viral RNA was present in the exosomes from TMEV-infected exosomes similar to microglia infected with TMEV (Figure 2). Since TMEV is a small positive-strand RNA virus, we wanted to determine whether the entire coding region of TMEV was present in the exosomes. Primers were used to generate a long piece which includes the coding region of the viral genome. Exosomes secreted from TMEV-infected microglia contained the entire coding region for TMEV similar to TMEV-infected microglia (Figure 2).

Since the exosomes secreted from TMEV-infected microglia contain viral RNA and exosomes can be taken up by other cells, we wanted to determine whether exosomes from TMEV-infected microglia can be taken up by uninfected microglia, bystander cells, and transfer the viral RNA. First, exosomes from TMEV-infected microglia were isolated and labeled with green florescence before being place on uninfected microglia to determine whether the exosomes were taken up by the uninfected microglia (Figure 2). Next, the RNA inside the exosomes isolated from TMEV-infected microglia was labeled with florescence dye before exosomes were placed on bystander microglia (Figure 2). The RNA from the TMEV-infected exosomes can be observed inside the cytoplasm of the bystander microglia and over time can be observed spreading around in the cytoplasm of the bystander microglia. To further determine whether the viral RNA was transferred to uninfected bystander microglia, the exosomes from the TMEV-infected microglia were added to the bystander microglia, and after 24 h, the bystander microglia were lysed and analyzed for viral RNA inside the cells. The bystander microglia contained viral RNA, although at lower levels compared to microglia directly infected with TMEV. To ensure the viral RNA was inside the exosomes, the exosomes isolated from TMEV-infected microglia were treated with RNAse or proteinase cocktail after isolation and then analyzed for viral RNA. These treated exosomes had similar levels of viral RNA as untreated exosomes indicating viral RNA was inside the exosomes. Furthermore, when these treated exosomes were placed on bystander microglia, the microglia contained similar levels of viral RNA after 24 h compared to untreated exosomes from TMEV-infected microglia. Finally, we wanted to determine whether the viral RNA that was transferred by the exosomes to the bystander microglia was able replicate in the recipient cells. The exosomes were isolated from TMEV-infected microglia and placed on bystander microglia for 4 h, the exosomes were removed and the cells were washed. The bystander microglia were incubated an additional 0, 4, 8, or 20 h before being lysed and analyzed for viral RNA. The viral RNA in the bystander microglia increased over time indicating viral replication occurred in the recipient cells. These results show that exosomes secreted by TMEV-infected microglia contain viral RNA that can be transferred to uninfected cells.

### Exosomes Secreted by Virus-Infected Microglia Activate Bystander CNS Cells

**FIGURE 3:**
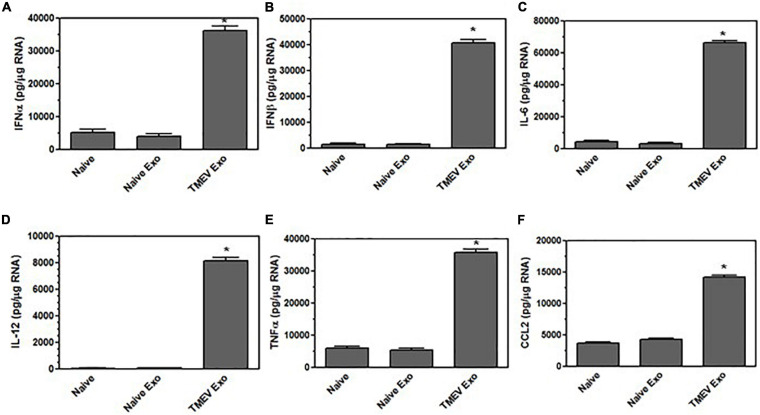
Exosomes from TMEV-infected microglia activate bystander microglia to express type I interferons and pro-inflammatory cytokines. Exosomes were isolated from TMEV-infected microglia or uninfected (naïve) microglia (100 μg) and placed on naive microglia (1 × 10^6^) for 24 h. Microglia were lysed, RNA isolated, converted to cDNA, and analyzed by real time PCR for expression of IFNα **(A)**, IFNβ **(B)**, IL-6 **(C)**, IL-12 **(D)**, TNFα **(E)**, and CCL2 **(F)**. Significant difference was determined by the one way ANOVA and Bonferroni’s multiple comparison test (*p < 0.001) based on unstimulated microglia. These are representative graphs from one experiment of five independent repeated experiments.

We have previously shown that microglia become activated to express innate immune cytokines, including pro-inflammatory cytokines and chemokines, during TMEV infection (Olson et al., 2001). We wanted to determine whether the exosomes secreted by the TMEV-infected microglia could activate uninfected bystander microglia to express cytokines and chemokines. Exosomes were isolated from TMEV-infected microglia and placed on uninfected bystander microglia. After 24 h, the recipient microglia were analyzed for expression of cytokines and chemokines. Bystander microglia exposed to exosomes from TMEV-infected microglia increased the expression of type I interferons, IFNα and IFNβ, cytokines, IL-6, IL-12, and TNFα, and chemokines, CCL2, compared to bystander microglia exposed to exosomes from uninfected microglia (Figure 3). The amount of exosomes added to the bystander microglia (100 μg per 1 × 10^6^ cells) was determined based on a dose response to varying amounts of exosomes. Approximately 200 μg of exosomes are isolated from 1 × 10^6^ TMEV-infected microglia (Supplementary Figure 2). Furthermore, exosomes secreted from TMEV-infected microglia that were treated with RNAse or proteinase cocktail prior to their addition to the bystander microglia showed a similar activation of bystander microglia as compared to untreated exosomes (Supplementary Figure 2). These results show that the contents inside the exosomes are activating the bystander microglia to increase expression of pro-inflammatory cytokines and chemokines. To determine activation time course, exosomes were isolated from TMEV-infected microglia and added to bystander microglia. After 4 h, the microglia cultures were washed to remove any exosomes that were not taken up. The microglia were incubated for an additional 0, 4, 8, or 20 h and then lysed for analysis of expression of cytokines and chemokines. The bystander microglia increased expression of cytokines and chemokines over time (Supplementary Figure 2). These results show that exosomes secreted by TMEV-infected microglia activate uninfected bystander microglia to secrete innate immune cytokines and chemokines.

**FIGURE 4:**
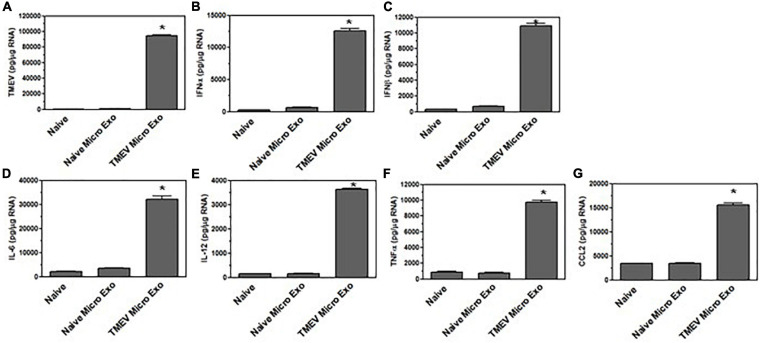
Exosomes from TMEV-infected microglia activate bystander astrocytes. Exosomes were isolated from uninfected (naïve) microglia or TMEV-infected microglia and placed on naive astrocytes for 24 h. Astrocytes were lysed, RNA isolated, converted to cDNA, and analyzed by real time PCR for expression of TMEV **(A)**, IFNα **(B)**, IFNβ **(C)**, IL-6 **(D)**, IL-12 **(E)**, TNFα **(F)**, and CCL2 **(G)**. Significant difference was determined by the one way ANOVA and Bonferroni’s multiple comparison test (*p < 0.001) based on unstimulated microglia. These are representative graphs from one experiment of four independent repeated experiments.

**FIGURE 5:**
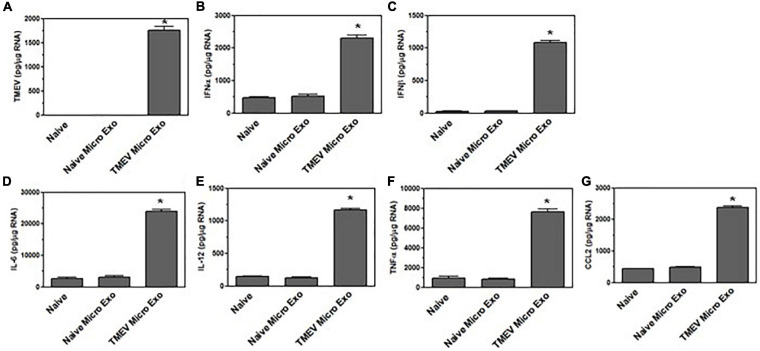
Exosomes from TMEV-infected microglia activate bystander neurons. Exosomes were isolated from uninfected (naïve) microglia or TMEV-infected microglia and placed on naive neurons for 24 h. Neurons were lysed, RNA isolated, converted to cDNA, and analyzed by real time PCR for expression of TMEV **(A)**, IFNα **(B)**, IFNβ **(C)**, IL-6 **(D)**, IL-12 **(E)**, TNFα **(F)**, and CCL2 **(G)**. Significant difference was determined by the one way ANOVA and Bonferroni’s multiple comparison test (*p < 0.001) based on unstimulated microglia. These are representative graphs from one experiment of three independent repeated experiments.

The CNS has several resident cells, including astrocytes and neurons, which could also take up exosomes secreted by microglia. Thus, exosomes isolated from TMEV-infected microglia were placed on uninfected bystander astrocytes. After 24 h, the astrocytes were examined for viral RNA and were also analyzed for the expression of innate immune cytokines and chemokines (Figure 4). Exosomes from TMEV-infected microglia transferred viral RNA to the bystander astrocytes and activated the astrocytes to express type I interferons, IFNα and IFNβ, as well as increase the expression of cytokines, IL-6, IL-12, and TNFα, and chemokines, CCL2. Similarly, exosomes from TMEV-infected microglia were placed on uninfected bystander neurons. After 24 h, the neurons were examined for viral RNA and for expression of cytokines and chemokines (Figure 5). Exosomes from TMEV-infected microglia transferred viral RNA to neurons and activated the neurons to express type I interferons, IFNα and IFNβ, cytokines, IL-6, IL-12, and TNFα, and chemokines, CCL2, compared to exosomes from uninfected microglia. These results show that exosomes secreted by TMEV-infected microglia activate expression of innate immune cytokines and chemokines in uninfected bystander CNS resident cells, astrocytes and neurons.

### Bystander Microglia Are Activated by the Viral RNA in Exosomes From TMEV-Infected Microglia

**FIGURE 6:**
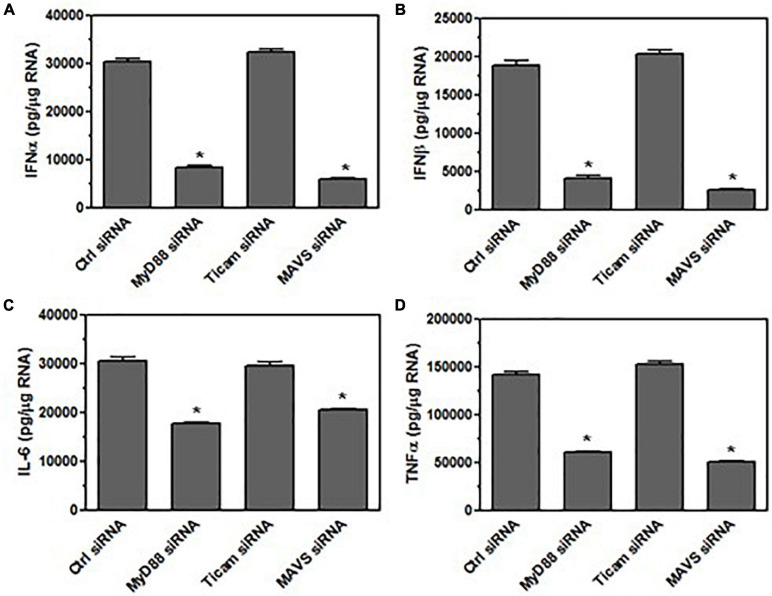
Bystander microglia are activated by viral RNA in exosomes from TMEV-infected microglia via innate immune receptors. Microglia were transfected with siRNA for MyD88, TICAM-1, MAVS, or control (6 h). Exosomes were isolated from TMEV-infected microglia and placed on the transfected microglia for 24 h. Microglia were lysed, RNA isolated, converted to cDNA, and analyzed by real time PCR for expression of IFNα **(A)**, IFNβ **(B)**, IL-6 **(C)**, and TNFα **(D)**. Significant difference was determined by the one way ANOVA and Bonferroni’s multiple comparison test (*p < 0.001) based on control siRNA transfected microglia. These are representative graphs from one experiment of three independent repeated experiments.

Since the exosomes contain viral RNA and viral RNA has been shown to be recognized by innate immune receptors to activate the innate immune response, we wanted to determine whether the viral RNA in the exosomes was activating the bystander microglia. Since TMEV is a single stranded RNA virus, the innate immune receptors that recognize single stranded RNA include TLR7 which signals through MyD88 (Nair and Diamond, 2015). Double stranded RNA is recognized by TLR3 which signals through TLR adaptor molecule 1 (Ticam1) to induce expression of type I interferons. Double stranded RNA is also recognized by melanoma differentiation-associated protein 5 (MDA5) which signals through MAVS to induce expression of type I interferons and cytokines (Nair and Diamond, 2015). Thus, microglia were silenced for MyD88, Ticam1, or MAVS prior to incubation with exosomes from TMEV-infected microglia (Figure 6). The silencing of the specific proteins was conducted with a pool of four siRNA to the target protein that reduces the expression of the target protein by 85–95% (Supplementary Figure 3). After 24 h, the microglia were analyzed for expression of type I interferons and pro-inflammatory cytokines. Microglia that were silenced for MyD88 and MAVS had greatly reduced expression of IFNα and IFNβ after exposure to TMEV-infected exosomes while there was no difference in type I interferon in microglia silenced for Ticam1. In addition, microglia silenced for MyD88 and MAVS also had reduced expression of cytokines, IL-6 and TNFα, after exposure to TMEV-infected exosomes. These results show that MyD88 and MAVS which are signaling pathways in the innate immune response to viral RNA were important for activating microglia to express type I interferons and pro-inflammatory cytokines after taking up exosomes secreted by TMEV-infected microglia.

### Microglia Secrete Exosomes During TMEV Infection in Mice That Contain Viral RNA and Activate Bystander CNS Cells

**FIGURE 7:**
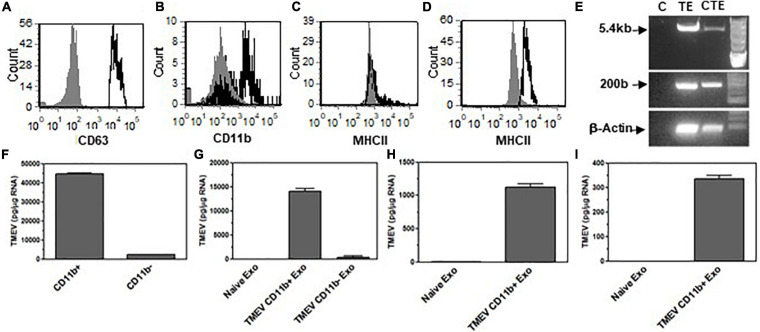
Exosomes secreted by microglia during TMEV infection of mice contain viral RNA that can be transferred to bystander CNS cells. Exosomes were isolated from the brains of TMEV-infected mice at day 2 post-infection (three mice per group). The exosomes were labeled with fluorescently labeled antibodies for CD63, CD11b, and MHC class II. The exosomes were analyzed by flow cytometry for CD63 **(A)** and CD11b **(B)** and MHC class II in CD11b^–^ exosomes **(C)** and CD11b^+^ exosomes **(D)** with the specific antibodies in black lines and isotype control antibodies in gray. **(E)** Mice were infected or mock infected with TMEV. At 2 days post-infection, exosomes were isolated from the brains and sorted for CD11b^+^ exosomes. RNA was isolated from the CD11b^+^ exosomes from mock infected mice (C) and TMEV-infected mice (TE). RNA was converted to cDNA, and used in PCR analysis with primers for TMEV long (5.4 kbp) or short (200 bp) products or with primers for β-actin. Microglia were incubated with CD11b^+^ exosomes isolated from the brains of TMEV-infected mice (lane CTE). The cells were lysed 24 h later and RNA isolated, converted to cDNA and used in PCR analysis. **(F)** At day 2 post-infection, exosomes were isolated from TMEV-infected mice brains and sorted into CD11b^+^ and CD11b^–^ exosomes. The exosomes were analyzed for TMEV using real time PCR. **(G–I)** The exosomes isolated from TMEV-infected mice brains were sorted into CD11b^+^ and CD11b^–^ exosomes and placed on unstimulated cultures of microglia, astrocytes, and neurons. After 24 h, the cells were lysed, RNA isolated, converted to cDNA and analyzed by real time PCR for TMEV in microglia **(G)**, astrocytes **(H)**, and neurons **(I)**. These are representative graphs from one experiment of four independent repeated experiments.

Microglia *in vitro* data showed that microglia infected with TMEV secrete exosomes that contain viral RNA and can activate bystander CNS cells, including microglia, astrocytes, and neurons to express type I interferons and pro-inflammatory cytokines. Next, we wanted to determine whether microglia in the brain of mice infected with TMEV also secrete exosomes that contain viral RNA during acute infection. SJL mice were infected with TMEV or mock infected, and at 2 days post-infection, the brain was removed and exosomes were isolated. The exosomes were analyzed by flow cytometry for expression of CD63, exosome marker, and CD11b, microglia (Figure 7). Approximately 60% of the exosomes isolated from the brain were derived from microglia as determined by CD11b expression, and this was consistent between TMEV-infected and mock-infected mice. Further analysis showed that MHC class II was on the surface of exosomes from TMEV-infected mice while exosomes from mock infected mice did not have MHC class II. These results are similar to MHC class II expression on microglia during TMEV infection in the brain. The exosomes isolated from the TMEV-infected mice were then sorted based on CD11b expression to isolate exosomes secreted from microglia. The CD11b^+^ exosomes from TMEV-infected mice contained viral RNA while CD11b^–^ exosomes had very little viral RNA. Most significantly, the CD11b^+^ exosomes contained the viral genome (Figure 7). The CD11b^+^ exosomes isolated from the brain of TMEV-infected mice did not contain viral particles as determined by plaque assay and mass spectrometric analysis (Supplementary Figure 1). Next, the CD11b^+^ exosomes from TMEV-infected mice were placed on uninfected microglia *in vitro* to determine whether the viral RNA could be transferred to bystander microglia. The CD11b^+^ exosomes from TMEV-infected mice transferred the viral RNA to bystander microglia including the viral genome, while the CD11b^–^ exosomes transferred very little viral RNA (Figure 7). Furthermore, CD11b^+^ exosomes from TMEV-infected mice were placed on bystander astrocytes and neurons *in vitro*. Similarly, the CD11b^+^ exosomes from TMEV-infected mice were able to transfer the viral RNA to the bystander astrocytes and neurons. These results show that microglia in the brain of TMEV infected mice secrete exosomes that contain viral RNA but not viral particles, and these exosomes transfer viral RNA to bystander, uninfected CNS cells.

**FIGURE 8:**
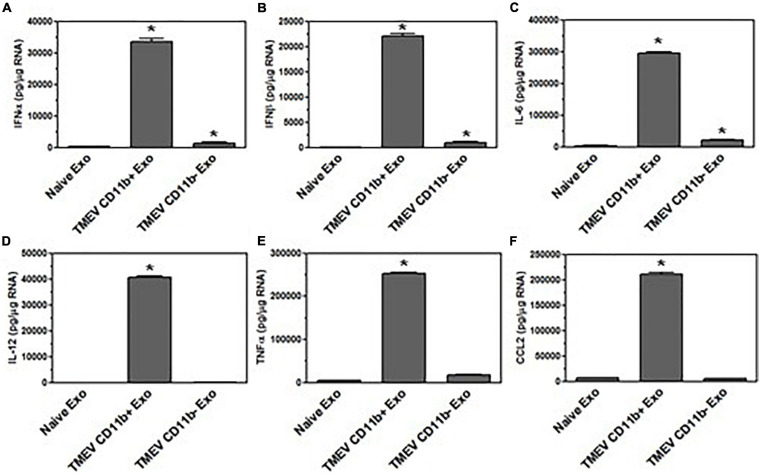
Exosomes secreted by microglia during TMEV infection in mice activate bystander microglia. Exosomes were isolated from the brains of TMEV-infected mice at 2 days post-infection (three mice per group). The exosomes from the brains of TMEV-infected mice and naïve mice were sorted for CD11b^+^ and CD11b^–^ exosomes which were then placed on unstimulated microglia. After 24 h, the microglia were lysed, RNA isolated, converted to cDNA, and used in real time PCR with primers for IFNα **(A)**, IFNβ **(B)**, IL-6 **(C)**, IL-12 **(D)**, TNFα **(E)**, and CCL2 **(F)**. Significant difference was determined by the one way ANOVA and Bonferroni’s multiple comparison test (*p < 0.001) based on microglia that were incubated with exosomes secreted by microglia in naive mice. These are representative graphs from one experiment of four independent repeated experiments.

**FIGURE 9:**
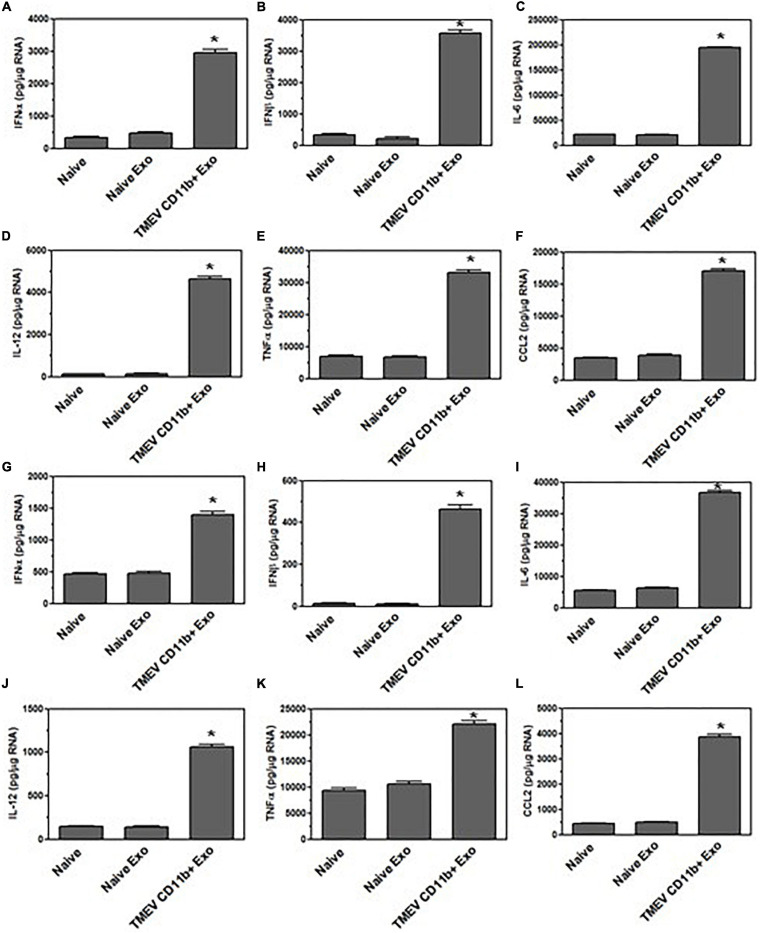
Exosomes secreted by microglia during TMEV infection in mice activate bystander CNS cells. Exosomes were isolated from the brains of TMEV-infected mice at 2 days post-infection or from naïve mice (three mice per group). The exosomes were sorted for CD11b^+^ exosomes which were placed on unstimulated astrocytes and neurons. After 24 h, the astrocytes **(A–F)** and neurons **(G–L)** were lysed, RNA isolated, converted to cDNA, and used in real time PCR with primers for IFNα **(A,G)**, IFNβ **(B,H)**, IL-6 **(C,I)**, IL-12 **(D,J)**, TNFα **(E,K)**, and CCL2 **(F,L)**. Significant difference was determined by the one way ANOVA and Bonferroni’s multiple comparison test (*p < 0.001) based on naive astrocytes or neurons. These are representative graphs from one experiment of three independent repeated experiments.

Since the exosomes secreted from TMEV-infected microglia *in vitro* activated bystander microglia, we wanted to determine whether the exosomes secreted by microglia in mice during TMEV infection could activate uninfected, bystander microglia. The CD11b^+^ exosomes isolated from the brains of TMEV-infected mice at 2 days post-infection were placed in culture with uninfected bystander microglia. After 24 h, the microglia were examined for expression of type I interferons, cytokines, and chemokines (Figure 8). The microglia that were incubated with CD11b^+^ exosomes from TMEV-infected mice increased the expression of type I interferons, IFNα and IFNβ, cytokines, IL-6, IL-12, and TNFα, and chemokines, CCL2. Meanwhile, CD11b^–^ exosomes isolated from TMEV-infected mice only slightly increased the expression of type I interferons and IL-6 in bystander microglia. Finally, CD11b^+^ exosomes isolated from naïve mice did not activate microglia to secrete type I interferons, cytokines, or chemokines. Similarly, when CD11b^+^ exosomes from TMEV-infected mice were transferred to bystander astrocytes and neurons, the CD11b^+^ exosomes increased the expression of type I interferons, cytokines, and chemokines in the bystander cells (Figure 9). These results show that CD11b^+^ exosomes secreted in the brain during TMEV infection in mice activate bystander microglia, astrocytes, and neurons to express type I interferons, IFNα and IFNβ, cytokines, IL-6, IL-12, TNFα, and chemokines, CCL2.

**FIGURE 10:**
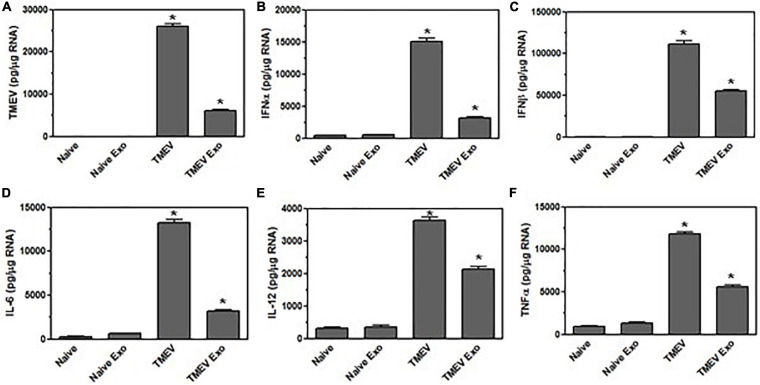
Exosomes secreted by microglia during TMEV infection in mice activate an inflammatory response in naïve mice. Exosomes were isolated from the brains of TMEV-infected mice at 2 days post-infection or from naïve mice (three mice per group). The exosomes were sorted for CD11b^+^ exosomes, and the CD11b^+^ exosomes were injected into the brain of naïve mice. At 2 days post-injection, the brains were removed from the mice (three mice per group), and RNA was isolated, converted to cDNA and used in real time PCR with primers for TMEV **(A)**, IFNα **(B)**, IFNβ **(C)**, IL-6 **(D)**, IL-12 **(E)**, and TNFα **(F)**. As comparison, the brains were removed from naïve mice or mice at 2 days post-TMEV infection, and RNA was isolated, converted to cDNA and used in real time PCR. Significant difference was determined by the one way ANOVA and Bonferroni’s multiple comparison test (*p < 0.001) based on the naïve mouse brain.

Since the exosomes secreted by microglia during TMEV infection in mice activated bystander cells *in vitro*, we wanted to determine whether these exosomes could activate an inflammatory response in the brain of a naïve mouse. CD11b^+^ exosomes were isolated from the brains of TMEV-infected mice at 2 days post-infection, the CD11b^+^ exosomes were injected into the brain of naïve mouse. At 2 days post-injection, the brains were removed and examined for expression of viral RNA (Figure 10). The mice that received the CD11b^+^ exosomes from the TMEV-infected mouse had viral RNA in the brain, although at a lower level compared to mice directly infected with TMEV. The brain from mice that received the CD11b^+^ exosomes did not have any viral particles detectable by plaque assay (Supplementary Figure 1). More interestingly, the CD11b^+^ exosomes isolated from TMEV-infected mice that were injected into the naïve mouse brains activated the expression of type I interferons, IFNα and IFNβ, as well as induced the expression of inflammatory cytokines, IL-6, IL-12, and TNFα (Figure 10). Although the levels of expression of the cytokines were less compared to directly infected mice, the levels of cytokines were significantly increased over the mice that had been injected with CD11b^+^ exosomes isolated from naïve mice. These results show that exosomes secreted by CD11b^+^ microglia during TMEV infection transfer viral RNA and promote an inflammatory immune response in uninfected mice.

## Discussion

Microglia are the immune resident population of the CNS. We have previously shown that microglia become persistently infected with TMEV. These studies show that TMEV-infected microglia secrete exosomes that can be taken up by bystander CNS cell including microglia, astrocytes, and neurons. The exosomes secreted by microglia during TMEV infection contain viral RNA which was transferred to uninfected CNS resident cells. We have previously shown that TMEV-infected microglia become activated to express type I interferons and pro-inflammatory cytokines and chemokines (Dal Canto, 1975; Lipton, 1975; Clatch et al., 1985; Lipton et al., 1986; Canto et al., 2000; Olson et al., 2001; Neville et al., 2002; Olson and Miller, 2004, 2009; Bowen and Olson, 2009). In these studies, we show that exosomes secreted from TMEV-infected microglia activate bystander microglia to express type I interferons and pro-inflammatory cytokines and chemokines via innate immune receptor recognition of viral RNA. Likewise, exosomes secreted from TMEV-infected microglia activate other CNS bystander cells including astrocytes and neurons to express type I interferons and pro-inflammatory cytokines and chemokines. The results from the *in vitro* studies were very interesting but we wanted to determine whether exosomes secreted by microglia during TMEV infection in mice could have similar effects on bystander cells. These studies show that exosomes secreted by microglia during TMEV infection in mice contain viral RNA but do not contain viral particles or viral proteins. The exosomes secreted by microglia in the brain during early TMEV infection activated bystander microglia, astrocytes, and neurons to secrete type I interferons and pro-inflammatory cytokines. Most importantly, exosomes secreted by microglia during TMEV infection in mice induced an inflammatory immune response when injected into naïve mice. These results show that TMEV-infected microglia secrete exosomes that not only transfer viral RNA to bystander uninfected CNS cells but also activate bystander cells to express pro-inflammatory cytokines and chemokines associated with neuroinflammation.

Following TMEV infection in SJL mice, infectious virus loads are very high in the brain for the first 1–3 days post-infection followed by a rapid spread to the spinal cord. The virus remains persistent in both the brain and spinal cord throughout the lifetime of the animal (Lipton et al., 1995). Microglia have been shown to be the persistently infected cells during TMEV infection, however, microglia produce very few infectious viral particles during infection (Trottier et al., 2001). Since microglia play an important role in the persistent virus infection, these studies focused on TMEV-infected microglia. We wanted to determine whether TMEV-infected microglia secrete exosomes that may contain viral products. In order to isolate exosomes that excluded viral particles, we used a method to isolate exosomes that does not include high speed centrifugation which would pellet the viral particles. TMEV is a non-enveloped virus around 40–50 nm in size. Our method yielded purified exosomes as verified by TEM staining for morphology, as verified for CD63 expression by flow cytometry and mass spectrometry, and as verified for particle size by Nanosight (120 nm). The isolated exosomes did not contain viral particles as verified by TEM staining which showed no viral particles isolated with the exosomes and no viral particles in exosomes, by plaque assays which showed no infectious particles isolated with the exosomes and no viral particles in exosomes, and by mass spectrometry which showed no viral proteins in exosomes. Since the exosomes do not contain viral particles, we wanted to determine whether the exosomes from TMEV-infected microglia contain viral RNA. TMEV is a small positive single-stranded RNA virus. Interestingly, the exosomes secreted from TMEV-infected microglia contained not only viral RNA but the whole viral coding region. To further determine whether microglia were secreting exosomes during TMEV infection in mice, we isolated exosomes secreted by microglia in the brains of TMEV infected mice at 2 days post-infection. The exosomes secreted by microglia (CD11b^+^) during TMEV infection in the brain contain viral RNA, including the entire TMEV genome. Meanwhile, the exosomes that were secreted by other CNS cells (CD11b^–^) in the brain during TMEV infection contained little or no viral RNA. The exosomes secreted by microglia during TMEV infection transferred the viral RNA to uninfected microglia, and the viral RNA replicated in the recipient cells. Similarly, exosomes secreted by microglia during TMEV infection transferred viral RNA to uninfected bystander astrocytes and neurons. These results show that exosomes secreted by microglia during TMEV infection can transfer viral RNA to uninfected cells independent of viral particles. Furthermore, the viral RNA transferred by the exosomes replicated in the recipient bystander cells. Thus, exosomes transport viral RNA between cells during TMEV infection which may be a mechanism to evade virus-specific antibodies directed toward viral particles and enable virus persistence.

Microglia infected with TMEV and microglia in the brain of mice infected with TMEV produce type I interferons which have a direct affect on the immune response during TMEV infection (Olson and Miller, 2009). In the current studies, the exosomes secreted by microglia during TMEV infection activated uninfected bystander microglia to express of type I interferons, IFNα and IFNβ. We have previously shown that microglia express several innate immune receptors that recognize viral RNA (Olson and Miller, 2004). Since TMEV is a single stranded RNA virus, the innate immune receptors in microglia, including TLR3, TLR7, and MDA5, could be recognizing the RNA in the exosomes secreted from TMEV-infected microglia. Each of these innate immune receptors activates a distinct signaling pathway to promote transcription of type I interferons. Silencing MyD88 which is activated by TLR7 or silencing MAVS which is activated by MDA5 in microglia exposed to exosomes secreted by microglia during TMEV infection reduced the expression of type I interferons. These results show that viral RNA in the exosomes was recognized by innate immune receptors in the bystander microglia which activated the expression of type I interferons. In addition, exosomes secreted by microglia during TMEV infection also activated the expression of type I interferons in bystander astrocytes and neurons. The expression levels of type I interferons in astrocytes and neurons were lower compared to expression by microglia. Type I interferons can have direct anti-viral activity, thus, low level expression of type I interferons in these bystander cells may protect the cells from a damaging virus infection, especially for neurons.

Microglia infected with TMEV become activated to express pro-inflammatory cytokines, chemokines, and effector molecules associated with neuroinflammation (Olson et al., 2001). The exosomes secreted by microglia during TMEV infection also induced the expression of pro-inflammatory cytokines, IL-6, IL-12, and TNFα, and chemokines, CCL2, in bystander uninfected microglia. The expression of cytokines and chemokines was reduced when innate immune signaling pathways involved in recognition of viral RNA were silenced, albeit, the expression was not completely reduced to naïve levels. This suggests that some of the increased expression of cytokines and chemokines is induced by the innate immune response to viral RNA. However, exosomes secreted by microglia during TMEV infection contain many other components, including miRNA and proteins, which may be also be activating the expression of cytokines and chemokines in the bystander cells. However, this difference in expression from naïve levels may also be due to siRNA not completely reducing expression levels (85–95% reduction). Further, the exosomes secreted by microglia during TMEV infection also activated bystander astrocytes and neurons to express cytokines and chemokines. Meanwhile, exosomes secreted by other cell types in the CNS (CD11b^–^) during TMEV infection contained low levels of viral RNA and were unable to activate bystander microglia. These results show that exosomes secreted by microglia during TMEV infection activate bystander uninfected cells to express pro-inflammatory cytokines and chemokines which may contribute to neuroinflammation during infection.

We have previously shown that microglia become activated following TMEV infection to increase the expression of co-stimulatory molecules and MHC class II (Olson et al., 2001). Interestingly, exosomes secreted by microglia during TMEV infection had increased levels of co-stimulatory molecules, CD80, CD86, CD40, on the surface compared to exosomes secreted by microglia during mock infection. Further, exosomes secreted by microglia during TMEV infection have MHC class II while exosomes secreted by uninfected microglia did not have MHC class II. These results show that exosomes have proteins on their surface which are similar to the activation state of the cells from which they are derived. Interestingly, about 60% of the exosomes isolated from the brain of TMEV infected mice were CD11b^+^ which suggests microglia are the predominant cell type secreting exosomes in the CNS but also suggests that other CNS cells secrete exosomes which may have an effect during virus infection. For this reason, these studies isolated CD11b^+^ exosomes to focus on exosomes secreted from microglia and compared them to CD11b^–^ exosomes which were secreted by other CNS cells.

Theiler’s murine encephalomyelitis virus infection in mice leads to neuroinflammation which contributes to the development of demyelinating disease. In these studies, exosomes secreted by microglia during TMEV infection were shown to activate bystander uninfected microglia, astrocytes, and neurons to express pro-inflammatory cytokines and chemokines associated with neuroinflammation. Further, to determine the effect of exosomes on neuroinflammation in the brain, the exosomes secreted by microglia during TMEV infection in mice were injected into naïve mice. Interestingly, the exosomes secreted by microglia during TMEV infection promoted the expression of pro-inflammatory cytokines and chemokines in the naïve mice. In addition, the exosomes secreted by microglia during TMEV infection transferred viral RNA and induced the expression of type I interferons in the brain of the naïve mice. These results show that exosomes secreted by microglia during TMEV infection transfer viral RNA and promote neuroinflammation in naïve mice suggesting that exosomes may play a role in virus persistence and neuroinflammation during TMEV infection. These results suggest that exosomes secreted by microglia during virus infections may contribute to neuroinflammation associated with development of neurological diseases.

## Data Availability Statement

The data presented in the study are deposited in the University of Minnesota Repository (https://hdl.handle.net/11299/220690).

## Ethics Statement

The animal study was reviewed and approved by University of Minnesota IACUC.

## Author Contributions

JO designed and directed the study, conducted the experiments, analyzed the data, and wrote the manuscript. NL conducted the experiments and contributed to writing to the manuscript. Both authors contributed to the article and approved the submitted version.

## Conflict of Interest

The authors declare that the research was conducted in the absence of any commercial or financial relationships that could be construed as a potential conflict of interest.

## Publisher’s Note

All claims expressed in this article are solely those of the authors and do not necessarily represent those of their affiliated organizations, or those of the publisher, the editors and the reviewers. Any product that may be evaluated in this article, or claim that may be made by its manufacturer, is not guaranteed or endorsed by the publisher.

## Supplementary Material

Supplementary Figure 1 Exosomes isolated from TMEV-infected microglia do not contain infectious viral particles. Exosomes were isolated from TMEV-infected microglia or mock infected microglia. The exosomes were analyzed by Nanosight for particle size **(A)**. The supernatant removed from TMEV-infected microglia (TMEV Sup) or mock infected microglia (Mock Sup). The exosomes isolated from TMEV-infected microglia (TMEV Exo) or mock infected microglia (Mock Exo) were used in a plaque assay with BHK cells **(B)**. Plaque forming units (PFU) were determined per ml of starting supernatant. The brain was removed from TMEV-infected mice at 2 days post-infection (TMEV brain) or from mock infected mice (Mock brain) and homogenized. The exosomes were isolated from the brains of TMEV infected mice at 2 days post-infection and then sorted into CD11b^+^ exosomes (TMEV CD11b^+^ Exo) and CD11b^–^ exosomes (TMEV CD11b^–^ Exo) **(C)**. Exosomes were also isolated from mock infected mice brains (Mock Exo). The brain homogenates and isolated exosomes were used in a plaque assay and plaque forming units (PFU) were counted based on mg of starting brain tissue. Exosomes isolated from TMEV-infected microglia were placed on naïve microglia cultures for 4 h. The cells were washed to remove any exosomes that were not taken up and then incubated an additional 20 h. The supernatant was removed and used in plaque assay with BHK cells **(D)**. The CD11b^+^ and CD11b^–^ exosomes were isolated from the brains of mice at 2 days post-infection or from naïve mice. The exosomes were injected intracranially into naïve mouse brains. After 2 days, the brains were removed and homogenized to be used in a plaque assay with BHK cells **(E)**. Plaque forming units were calculated as described above.

Supplementary Figure 2 Exosomes from TMEV-infected microglia have contents that activate bystander microglia in dose dependent manner within 4 h. Exosomes were isolated from TMEV-infected microglia or mock infected microglia. The exosomes from TMEV-infected microglia were quantified and placed on unstimulated microglia (1 × 10^6^) at concentration of 200, 100, 20, or 2 μg for 24 h. Microglia were lysed, RNA isolated, converted to cDNA, and analyzed by real time PCR for expression of TMEV **(A)**, IFNβ **(B)**, and TNFα **(C)**. Exosomes were isolated from TMEV-infected microglia or mock infected microglia. The exosomes from TMEV-infected microglia were control treated, RNAse treated, or proteinase treated. The exosomes were then placed in culture with naïve microglia for 24 h. After 24 h, the microglia were lysed, RNA isolated, converted to cDNA, and analyzed by real time PCR for expression of IFNβ **(D)** and TNFα **(E)**. Exosomes were isolated from TMEV-infected microglia and placed on naïve microglia. After 4 h, the exosomes were removed and the cells were washed. The microglia were incubated for an additional 0, 4, 8, or 12 h before being lysed and analyzed by real time PCR for expression of IFNβ **(F)** and TNFα **(G)**. Significant difference was determined by the one way ANOVA and Bonferroni’s multiple comparison test (**p* < 0.001) based on unstimulated microglia. These are representative graphs from one experiment of four independent repeated experiments.

Supplementary Figure 3 Efficiency of siRNA in bystander microglia. Microglia were transfected with siRNA for MyD88, TICAM-1, MAVS, or control for 6 h. Microglia were lysed, RNA isolated, converted to cDNA, and analyzed by real time PCR for expression of MyD88 (control siRNA and MyD88 transfected microglia), Ticam-1 (control siRNA and Ticam-1 transfected microglia), or MAVS (control siRNA and MAVS transfected microglia). The percent expression was calculated for each primer pair based on the expression in control siRNA transfected microglia for that primer (100% expression).

Supplementary Table 1 Protein analysis in identification of exosomes.

## Abbreviations

CNS: central nervous system
TMEV: Theiler’s murine encephalomyelitis virus
MS: multiple sclerosis
PLP: proteolipid protein.
