# Supplementary figures and images for: Exosomes Secreted by Microglia During Virus Infection in the Central Nervous System Activate an Inflammatory Response in Bystander Cells
Source: Front Cell Dev Biol. 2021 Aug 13;9:661935. doi: 10.3389/fcell.2021.661935 (PMC8415116; doi:10.3389/fcell.2021.661935)

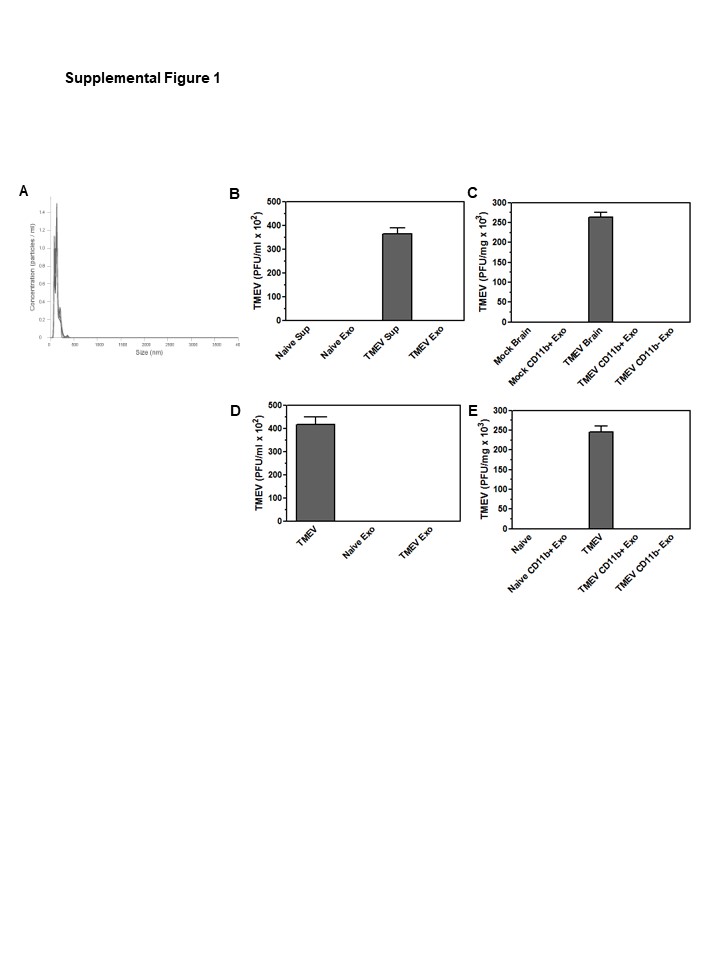

Supplement: Supplementary Figure 1 — Exosomes isolated from TMEV-infected microglia do not contain infectious viral particles. Exosomes were isolated from TMEV-infected microglia or mock infected microglia. The exosomes were analyzed by Nanosight for particle size (A). The supernatant removed from TMEV-infected microglia (TMEV Sup) or mock infected microglia (Mock Sup). The exosomes isolated from TMEV-infected microglia (TMEV Exo) or mock infected microglia (Mock Exo) were used in a plaque assay with BHK cells (B). Plaque forming units (PFU) were determined per ml of starting supernatant. The brain was removed from TMEV-infected mice at 2 days post-infection (TMEV brain) or from mock infected mice (Mock brain) and homogenized. The exosomes were isolated from the brains of TMEV infected mice at 2 days post-infection and then sorted into CD11b+ exosomes (TMEV CD11b+ Exo) and CD11b– exosomes (TMEV CD11b– Exo) (C). Exosomes were also isolated from mock infected mice brains (Mock Exo). The brain homogenates and isolated exosomes were used in a plaque assay and plaque forming units (PFU) were counted based on mg of starting brain tissue. Exosomes isolated from TMEV-infected microglia were placed on naïve microglia cultures for 4 h. The cells were washed to remove any exosomes that were not taken up and then incubated an additional 20 h. The supernatant was removed and used in plaque assay with BHK cells (D). The CD11b+ and CD11b– exosomes were isolated from the brains of mice at 2 days post-infection or from naïve mice. The exosomes were injected intracranially into naïve mouse brains. After 2 days, the brains were removed and homogenized to be used in a plaque assay with BHK cells (E). Plaque forming units were calculated as described above. [file Image_1.jpeg]

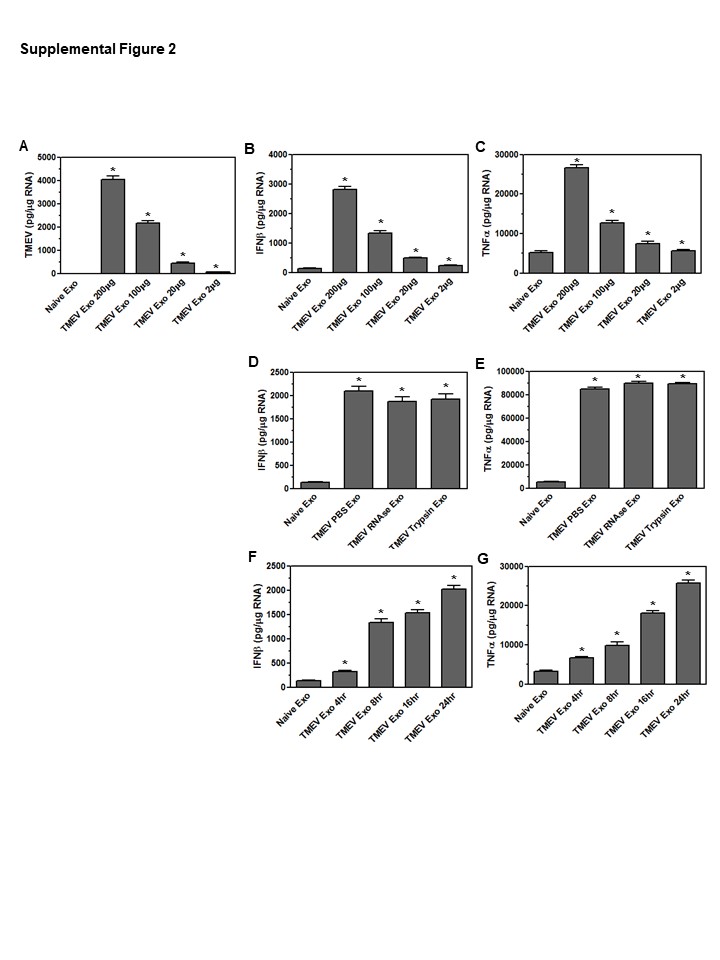

Supplement: Supplementary Figure 2 — Exosomes from TMEV-infected microglia have contents that activate bystander microglia in dose dependent manner within 4 h. Exosomes were isolated from TMEV-infected microglia or mock infected microglia. The exosomes from TMEV-infected microglia were quantified and placed on unstimulated microglia (1 × 10^6^) at concentration of 200, 100, 20, or 2 μg for 24 h. Microglia were lysed, RNA isolated, converted to cDNA, and analyzed by real time PCR for expression of TMEV (A), IFNβ (B), and TNFα (C). Exosomes were isolated from TMEV-infected microglia or mock infected microglia. The exosomes from TMEV-infected microglia were control treated, RNAse treated, or proteinase treated. The exosomes were then placed in culture with naïve microglia for 24 h. After 24 h, the microglia were lysed, RNA isolated, converted to cDNA, and analyzed by real time PCR for expression of IFNβ (D) and TNFα (E). Exosomes were isolated from TMEV-infected microglia and placed on naïve microglia. After 4 h, the exosomes were removed and the cells were washed. The microglia were incubated for an additional 0, 4, 8, or 12 h before being lysed and analyzed by real time PCR for expression of IFNβ (F) and TNFα (G). Significant difference was determined by the one way ANOVA and Bonferroni’s multiple comparison test (*p < 0.001) based on unstimulated microglia. These are representative graphs from one experiment of four independent repeated experiments. [file Image_2.jpeg]

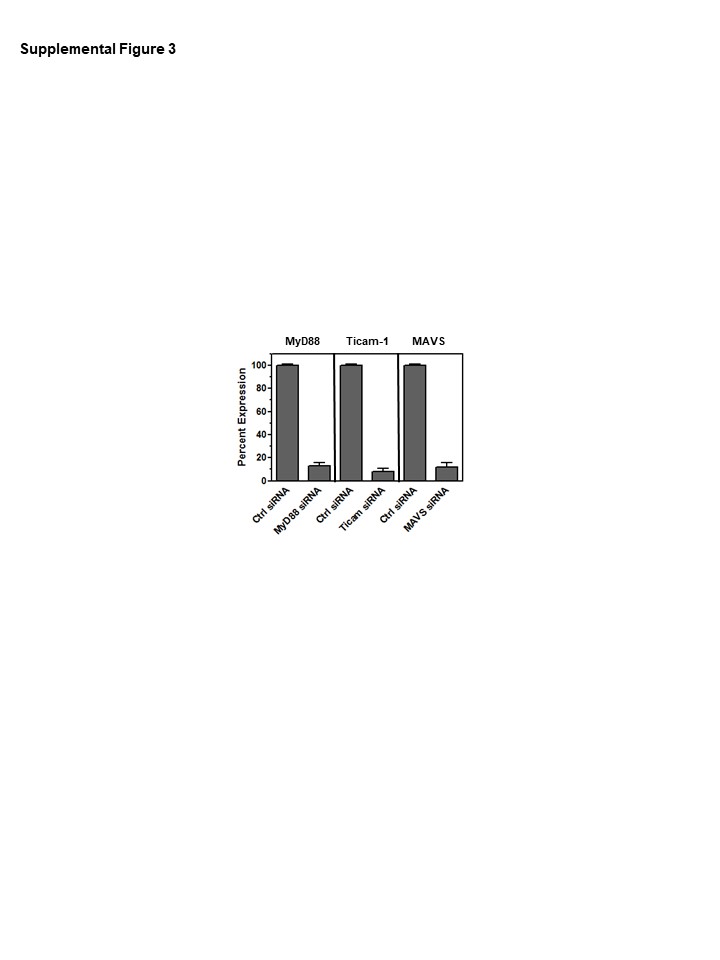

Supplement: Supplementary Figure 3 — Efficiency of siRNA in bystander microglia. Microglia were transfected with siRNA for MyD88, TICAM-1, MAVS, or control for 6 h. Microglia were lysed, RNA isolated, converted to cDNA, and analyzed by real time PCR for expression of MyD88 (control siRNA and MyD88 transfected microglia), Ticam-1 (control siRNA and Ticam-1 transfected microglia), or MAVS (control siRNA and MAVS transfected microglia). The percent expression was calculated for each primer pair based on the expression in control siRNA transfected microglia for that primer (100% expression). [file Image_3.jpeg]
