# Supplementary material for: Exosomes Secreted by Microglia During Virus Infection in the Central Nervous System Activate an Inflammatory Response in Bystander Cells
Source: Front Cell Dev Biol. 2021 Aug 13;9:661935. doi: 10.3389/fcell.2021.661935 (PMC8415116; doi:10.3389/fcell.2021.661935)
Supplement: Supplementary Table 1 — Protein analysis in identification of exosomes. [file Table_1.docx]

| **Supplemental Table I. Protein Analysis in Identification of Exosomes** | | |
| --- | --- | --- |
|  |  |  |
| **Proteins Present in Exosomes** |  | **Proteins Absent in Exosomes** |
| **CD63 antigen** |  | **Grp94 (HSP90B1)** |
| **Integrin alpha-1 (Itga1)** |  | **Clanexin (CANX)** |
| **CD81 antigen** |  | **Golgi (GM130)** |
| **CD9 antigen** |  | **cytochrome C (cytC)** |
| **Tumor susceptibility gene 101 protein (Tsg101)** |  | **histones (HIST*H*)** |
| **Ras-related protein Rab-14 (Rab14)** |  | **Argonaute/RISC complex (AGO*)** |
| **Putative uncharacterized protein (Rab5c)** |  |  |
| **Ras-related protein Rab-5A (Rab5a)** |  | **TMEV viral capsid protein 3 (VP3)** |
| **Ras-related protein Rab-5B (Rab5b)** |  | **TMEV viral protein 3C** |
| **Ras-related protein Rab-2A (Rab2a)** |  | **TMEV viral protein 2C** |
| **Ras-related protein Rab-10 (Rab10)** |  |  |
| **Ras-related protein Rab-18 (Rab18)** |  |  |
| **Ras-related protein Rab-7a (Rab7a)** |  |  |
| **Ras-related protein Rab-14 (Rab14)** |  |  |
| **Annexin A2 (Anxa2)** |  |  |
| **Cell adhesion molecule 3 (Cadm3)** |  |  |
| **BDNF/NT-3 growth factors receptor (Ntrk2)** |  |  |
| **Annexin A5 (Anxa5)** |  |  |
